# Identification of candidate gene associated with maize northern leaf blight resistance in a multi-parent population

**DOI:** 10.1007/s00299-024-03269-w

**Published:** 2024-07-03

**Authors:** Yaqi Bi, Fuyan Jiang, Xingfu Yin, Ranjan K. Shaw, Ruijia Guo, Jing Wang, Xingming Fan

**Affiliations:** Institute of Food Crops, Yunnan Academy of Agricultural Sciences, Kunming, China

**Keywords:** *Zea mays*, Northern leaf blight, Multi-parent population, Genome-wide association analysis, QTL mapping, CATETO germplasm

## Abstract

**Key message:**

QTL mapping combined with genome-wide association studies, revealed a potential candidate gene for  resistance to northern leaf blight in the tropical CATETO-related maize line YML226, providing a basis for marker-assisted selection of maize varieties

**Abstract:**

Northern leaf blight (NLB) is a foliar disease that can cause severe yield losses in maize. Identifying and utilizing NLB-resistant genes is the most effective way to prevent and control this disease. In this study, five important inbred lines of maize were used as parental lines to construct a multi-parent population for the identification of NLB-resistant loci. QTL mapping and GWAS analysis revealed that QTL *qtl_YML226_1*, which had the largest phenotypic variance explanation (PVE) of 9.28%, and SNP 5-49,193,921 were co-located in the CATETO-related line YML226. This locus was associated with the candidate gene *Zm00001d014471,* which encodes a pentatricopeptide repeat (PPR) protein. In the coding region of *Zm00001d014471*, YML226 had more specific SNPs than the other parental lines. qRT-PCR showed that the relative expressions of *Zm00001d014471* in inoculated and uninoculated leaves of YML226 were significantly higher, indicating that the expression of the candidate gene was correlated with NLB resistance. The analysis showed that the higher expression level in YML226 might be caused by SNP mutations. This study identified NLB resistance candidate loci and genes in the tropical maize inbred line YML226 derived from the CATETO germplasm, thereby providing a theoretical basis for using modern marker-assisted breeding techniques to select genetic resources resistant to NLB.

**Supplementary Information:**

The online version contains supplementary material available at 10.1007/s00299-024-03269-w.

## Introduction

Maize northern leaf blight (NLB), caused by the fungal pathogen *Exserhilum turcicum*, is a foliar disease that causes  serious yield losses worldwide (Zwonitzer et al. 2010; Yang et al. 2017). By identifying genes related to NLB resistance and transferring resistance genes to susceptible parental lines, maize yield loss can be significantly reduced. Quantitative trait locus (QTL) mapping and genome-wide association studies (GWAS) have been widely used to identify NLB resistance loci in maize(Kump et al. 2011; Zhang et al. 2012; Xu et al. 2014; Chen et al. 2021; Kibe et al. 2020). For instance, Balint-Kurti et al. (2010) detected two QTLs on chromosomes 2 and 4 in a high-generation recombinant inbred line (RIL) population derived from the cross Mo17 × B73, which explained 6.7% and 4.3% of phenotypic variance (PVE), respectively. Wang et al. (2018) identified 11 QTLs from cross Ye478 × Qi319, and identified *qNCLB7.02* as a consistent major resistant locus across multiple environments, with PVE ranging from 10.11% to 15.29%. Ranganatha et al. (2020) detected five NLB resistant QTLs with PVE ranging from 1.64% to 16.34% in 344 families of an F_2:3_ population derived from cross CML153 × SKV50. Ding et al. (2015) conducted GWAS for mean rating, high rating and area under the disease progress curve using single-marker and haplotype-based associations in a population of 999 maize inbred lines, and identified multiple SNPs associated with NLB across all 10 maize chromosomes. However, these studies primarily used temperate maize germplasms to identify NLB resistance loci, and very limited research on NLB resistance has been conducted using tropical maize germplasms.

Tropical maize grows under hot and humid climatic conditions and faces severe biotic and abiotic stresses. Therefore, the tropical maize germplasm retained more favorable variations during domestication, conferring stronger resistance to diseases than temperate maize. The CATETO germplasm, originating in South America, has been proven to exhibit high general combining ability and disease resistance in both temperate and tropical-subtropical environments, thus, it has been widely used in breeding programs (Santos et al. 2001; Subedi 2015; Fan et al. 2018; Hu et al. 2024). Therefore, identifying NLB resistant genes against maize NLB in populations derived from the tropical germplasm may be a good strategy for marker-assisted breeding.

In this study, five widely used elite maize inbred lines were used to construct a multi-parent population (MPP) with significant diversity in genetic background for NLB resistance. Among the parental lines, YML226, which exhibits NLB resistance, is a tropical maize inbred line selected from the CATETO-related germplasm. Given the high breeding value of the parents in this MPP, it is more likely to identify novel NLB-resistant genes suitable for practical breeding applications, thereby providing an important theoretical basis for the utilization of NLB resistance in maize molecular breeding programs.

## Materials and methods

### Plant material and field experimental design

In this study, the MPP was developed by crossing the common parental line Ye107, which exhibits moderate resistance to NLB, with four inbred lines (YML1218, Chang7-2, Q11, and YML226), each showing varying degrees of resistance or susceptibility to NLB. The MPP consists of 797 families belonging to four RIL subpopulations (RIL_YML1218, RIL_Chang7-2, RIL_Q11, and RIL_YML226). Among these, 199 families of RIL_YML1218 were obtained from the cross YML1218 × Ye107, 199 families of RIL_Chang7-2 were derived from the cross Chang7-2 × Ye107, 200 families of RIL_Q11 were obtained from the cross Q11 × Ye107, and 199 families of RIL_YML226 were obtained from the cross YML226 × Ye107. The pedigrees and ecotypes of the parents are listed in Table 1.

**Table 1:** Parental lines used in the MPP construction

Parents	Pedigree	Ecotypes	NLB score
Ye107	Selected from US inbred DeKalb XL80	Temperate	5
YML1218	Variation of K12 (Huangzaosi × Huaichun)	Temperate	7
Chang7-2	V59’HuangZaoSi	Temperate	7
Q11	Derived from US inbred	Temperate	9
YML226	(CML226/(CATETO DC1276/7619))F2-25–1-B-1	Tropical	3

In 2020, the MPP and its parental lines were planted and evaluated for NLB resistance in Jinghong (N27°27’, E100°25’), Yanshan (N23°62’, E104°33’), and Dehong (N24°43’, E98°59’) in Yunnan Province, China. Each field trial was arranged in a randomized complete block design with two replicates. The planting density was 0.8 m between plants and row length of 3 m, with single-row planting and 14 plants per row. Standard field management practices were followed during the trials.

### Culture and artificial inoculation of pathogen for NLB

Artificial inoculation of *Exserhilum turcicum* was conducted according to the method described by Ding et al. (2015). Pure cultures of the pathogen (serial number: BNCC150065) were purchased from the BeNa Culture Collection Co., Ltd. (Hebei, China), and grown on PDA medium and used to inoculate sterile sorghum grains to produce large volumes of the inoculum. Inoculated bottles containing infected sorghum grains were cultured at room temperature (25℃) for two weeks. The RILs of the MPP at the 6–8 leaf stage were inoculated by placing 20 grains of *E. turcicum*-colonized sorghum in the leaf whorl. Control plants were simultaneously inoculated with sterile and non-inoculated sorghum.

### Phenotypic data analysis

**Fig. 1:**
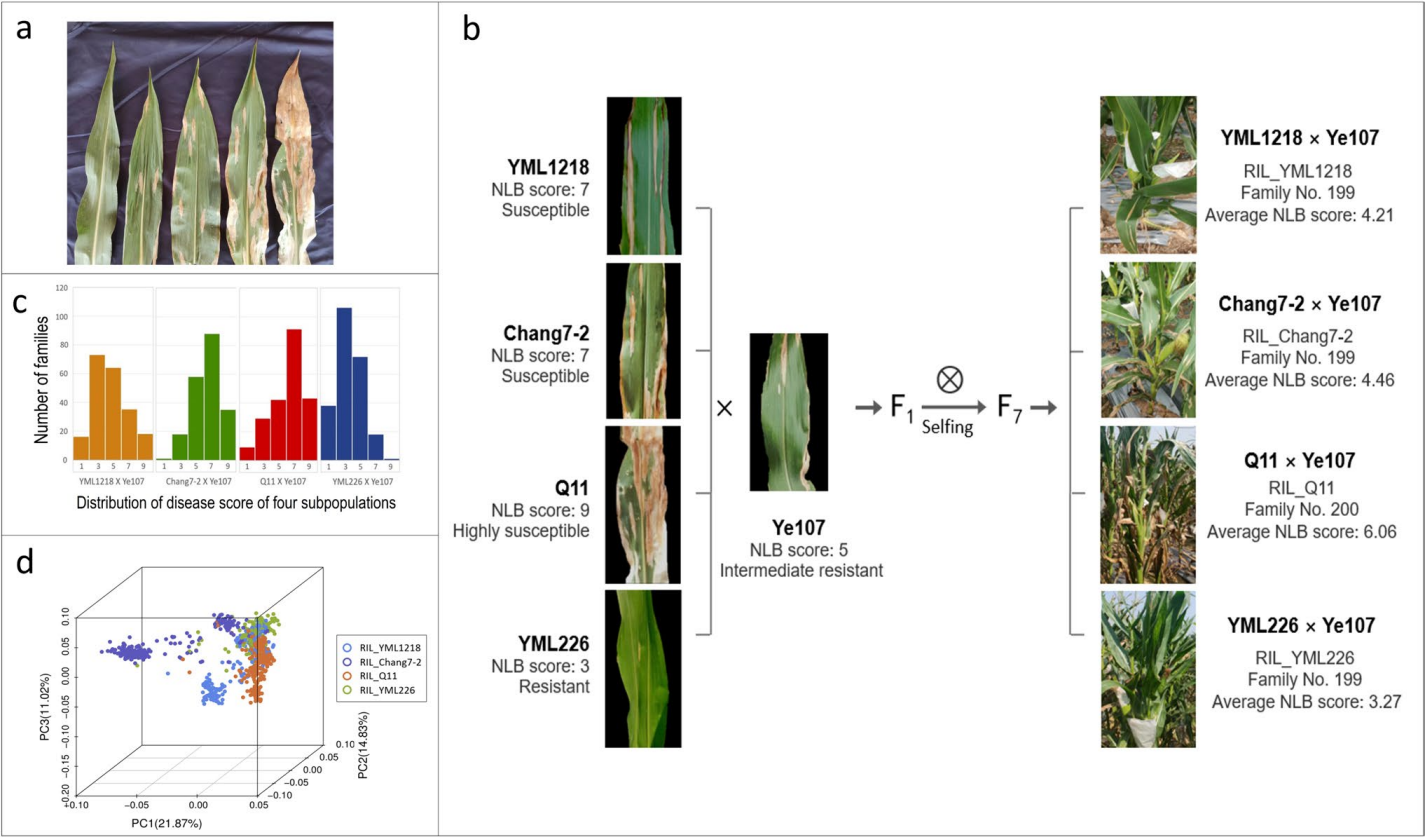
**a** Disease scores ranging from phenotypes of 1-9 used to evaluate NLB resistance. **b** The construction of the multi-parent population (MPP). Ye107 serves as the common parent crossed with the four parental lines. After seven cycles of selfing, the MPP was developed and evaluated for NLB resistance. **c** Frequency distributions of NLB resistance in the four RIL subpopulations. **d** Principal component analysis of the MPP. Blue, purple, orange, and green dots represent the families of YML1218 × Ye107, Change7-2 × Ye107, Q11 × Ye107 and YML226 × Ye107, respectively (color figure online)

After 30 d of pollination, phenotyping for NLB resistance was performed as described by Wang et al. (2014). The scoring method for NLB resistance proposed by Tao et al. (2015) ranges from 0 to 9, where 1 indicates high tolerance, 3 indicates resistance, 5 indicates moderate resistance, 7 indicates susceptibility, and 9 indicates high susceptibility (Fig. 1a). Ten plants (per family) grown in the middle of each row were selected and scored from 0 to 9. The average score of the ten plants was used as the score for that family. The ggplot2 package in R (v3.2.2) software was used to analyze the phenotypic data of each RIL in the MPP. The broad-sense heritability (H^2^) of the MPP was calculated using the GREML method by the GCTA software (Yang et al. 2010).

### Genotyping and principal component analysis

DNA was extracted from young leaves of each family of the MPP, and whole-genome resequencing was performed. DNA libraries were prepared according to standard procedures, and sequencing was performed using the Illumina HiSeq™ platform. Subsequently, the filtered reads were mapped to the maize reference genome B73_AGPv4 to identify SNP markers, which were annotated using the SNPeff software. SNP markers with a minor allele frequency (MAF) < 0.05 and a missing rate > 0.2 were filtered out. Principal component analysis (PCA) was conducted for the MPP using the GCTA software (Reverter and Fortes 2013).

### GWAS and QTL mapping of the RIL subpopulations

SNPs significantly associated with NLB resistance were identified by analyzing the phenotypic and genotypic data. All SNPs were filtered as described above. Three models, FarmCPU, general linear model (GLM), and mixed linear model (MLM), were used for GWAS. The rMVP (1.0.6) package in R was utilized for GWAS using the three models, with a threshold of α < 0.01 and Bonferroni correction based on the three principal components was applied during association analysis (Kusmec and Schnable 2018; Yin et al. 2021). The best model was selected based on the λ values of GWAS in the MPP, and then the selected model was applied to the MPP which consisted of 797 families, and for each RIL subpopulation that contained approximately 200 families (Shi et al. 2022). Candidate genes were screened within a 50 kb range upstream and downstream of the SNPs (Jiang et al. 2023; Bi et al. 2024). Linkage mapping was performed for each of the four RIL subpopulations, and SNPs showing no polymorphism between the two parental lines of each RIL subpopulation were filtered out. Bin markers were generated using the SNP binner and genetic maps were constructed using JoinMap and SMOOTH software in a two-step process (Van Os et al. 2005; Ooijen and Ooijen 2006). Independent LOD values ranging from 4-20 were used to group all markers in genome-wide for correcting their positions. The order of the markers in each genetic map was determined using the maximum-likelihood method. The Kosambi mapping function was used to convert the recombination frequencies to map the distances. Composite interval mapping (CIM) was conducted using R/QTL software to detect NLB resistant QTL with a threshold of *P* < 0.05, determined by 1000 random permutations (Broman et al. 2003).

### Epistatic and breeding value analysis of SNPs associated with NLB resistance

Epistatic effects associated with NLB resistant loci were analyzed in the RILs with the strongest positive effect. PLINK software was used for linear model regression analysis, with the formula y = u + g_i_ + g_j_ + g_i_g_j_, where y represents the NLB resistant score, g_i_ and g_j_ represent the main effects associated with markers i and j, and g_i_g_j_ represents the interaction effect between alleles at markers i and j, respectively. Significant positive epistatic results were obtained at *p* < 0.05.

The genomic estimated breeding value (GEBV) of MPP was calculated using the rr-BLUP package in R software. The best linear unbiased prediction method was used to calculate the relationship matrix using a mixed linear model for standardized genotype and phenotype data (Endelman 2011).

### Functional gene variation analysis

The genotypic data of the five parental lines of MPP were aligned with the reference genome, B73_AGPv4. Information of SNPs within the coding region and amino acid variations in candidate functional genes was extracted. The SNP and amino acid variation information were compared using DNAMAN, and MEME online software (https://meme-suite.org/meme/tools/meme) was used to predict the functional gene motifs.

### qRT-PCR and gene expression analysis

The Tiangen SuperReal PreMix Plus (SYBR Green) reagent kit (Tiangen, Beijing, China) was used for qRT-PCR to measure the relative expression of candidate gene in different parental lines, with a total reaction volume of 20 μL. Maize plants were infected with *Exserhilum turcicum* cultured sorghum grains, and the infected leaves were collected at 0.5, 1, 3, 6, 9, 12, 24, and 48 h after artificial inoculation. The expression of the candidate gene *Zm00001d014471* was quantified using the following primer sequences for three replicates: F-5’ TGAGCAGTGTCCGCAACA and R-5’ CCTCTTCAACCATCCCAGTA, GAPDH was used as the reference gene for qRT-PCR. The reaction program was set as follows: pre-denaturation at 95℃ for 3 min; denaturation at 95℃ for 20 s, annealing and extension at 58℃ for 30 s, for a total of 40 cycles. Fluorescence signals were detected during annealing and extension stages (60℃).

## Results

### Phenotyping and assessment of NLB resistance

In 2020, the MPP population was characterized to evaluate NLB resistance (Fig. 1b). The common parent Ye107 showed moderate resistance to NLB, whereas the other four parental lines exhibited resistance or susceptibility to NLB (Table 1). Among these, YML226, with a CATETO genetic background, demonstrated the strongest resistance to NLB. Correspondingly, RIL_YML226 subpopulation displayed the strongest resistance to NLB, with an average NLB score of 3.27. The parental line Q11 was highly susceptible to NLB and the RIL_Q11 subpopulation showed the highest susceptibility (average NLB score = 6.06). The other two subpopulations, RIL_YML1218 and RIL_Change7-2, exhibited similar resistance to NLB, with average NLB scores of 4.21 and 4.46, respectively (Fig. 1c). Analysis of variance (ANOVA) revealed significant differences in NLB scores among the RIL subpopulations, but no significant differences were observed between replicates and locations (Table 2). Broad-sense heritability (*H*^*2*^) of the MPP was 0.80 ± 0.05. ANOVA and *H*^*2*^ indicated that phenotypic variation in NLB resistance was primarily influenced by genetic factors.

**Table 2:** Analysis of variance for NLB resistance in four RIL subpopulations across three locations

	Df	Sum Sq	Mean Sq	F value	Pr(> F)
Replicates	1	4	4.2	0.9	0.342856
Populations	3	4481	1493.6	323.709	< 2e-16***
Locations	2	18	17.7	3.833	0.050339
Residuals	3415	15,757	4.6		

### PCA and testing of GWAS models

After filtering, a total of 18,189,449 SNPs were obtained through whole-genome resequencing of the RILs of the MPP. These SNPs were distributed across the 10 chromosomes of maize. Among these, chromosome 1 had the largest number of SNPs (2,467,471), while chromosome 10 had the lowest number of SNPs (1,200,136). The average marker density across the genome was 8.63/kb. Chromosome 4 exhibited the highest marker density (9.30/kb), whereas chromosome 10 had the lowest marker density (7.95/kb) (Supplementary Table 1).

Principal component analysis (PCA) results showed that the first three principal components accounted for 21.9%, 14.8%, and 11.0% of the total variance of the MPP, respectively (Fig. 1d). Generally, most families from the same RIL population clustered together, but a few families clustered with other RIL populations, likely due to the common male parent Ye107 (Fig. 1d). For GWAS analysis, the FarmCPU, MLM, and GLM models were used. The λ values of the three models were 1.08, 0.98, and 1.3, respectively, indicating that FarmCPU and MLM models were better suited for GWAS. Since FarmCPU demonstrated better control over false positives and false negatives compared to MLM, FarmCPU was selected for GWAS in this study (Liu et al. 2016; Kaler et al. 2020).

### GWAS analysis and QTL mapping of NLB resistance

**Fig. 2:**
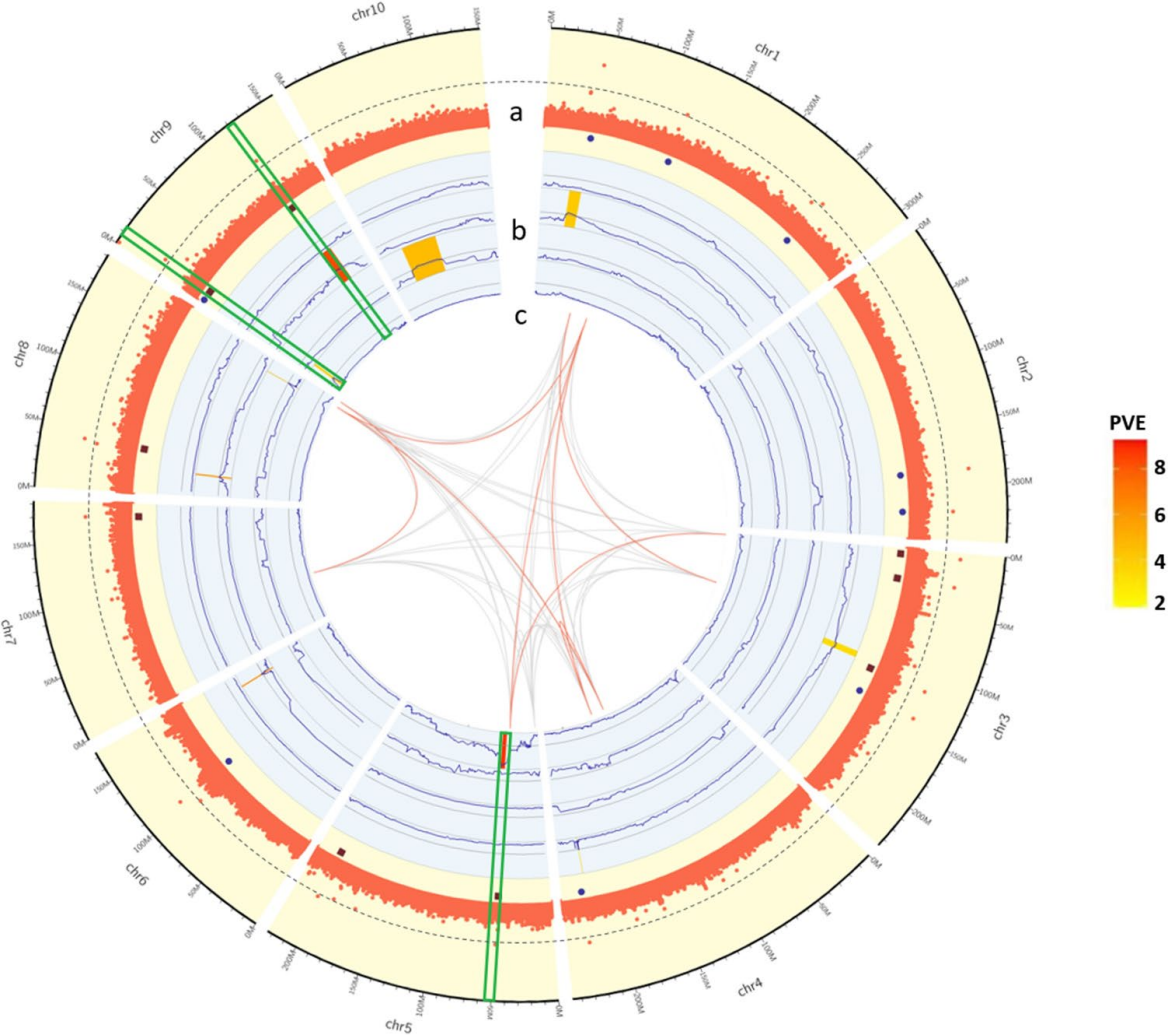
The identification of candidate SNPs and QTLs for NLB resistance. The green boxes represent the four overlapping intervals that were identified through both GWAS and QTL mapping. **a** Significant SNPs from GWAS analysis for NLB resistance in the MPP. The purple square represents SNPs with positive effect and the blue circle represents SNPs with negative effect. **b** The QTL mapping results and QTL effects on NLB resistance in four subpopulations. RIL_YML1218, RIL_Chang7-2, RIL_Q11 and RIL_YML226 are ordered from outside to inside. **c** SNPs with epistatic effect in the RIL_YML226 population. The red lines represent SNPs with significant epistatic effects (color figure online)

Through GWAS, 18 SNPs that were significantly associated with NLB resistance were detected in the MPP population (with a threshold of *P* < 1 × 10^–8^), and the *P*(-log10) values of these SNPs ranged from 8.50 to19.38 (Table 3, Fig. 2a). Among these, 11 SNPs were associated with 21 candidate genes (Supplementary Table 2). To further confirm the effects and origins of the SNPs that were significantly associated with NLB resistance, genetic linkage maps were constructed for the four RIL subpopulations (Fig. 2b). The bin markers used for the genetic map construction for the RIL_YML1218, RIL_Chang7-2, RIL_Q11, and RIL_YML226 subpopulations were 2075, 5258, 2459, and 3058, respectively. The map lengths of the four RIL subpopulations were 2451–3312 cM. During QTL mapping, two QTLs located on chromosomes 3 and 4 were detected in the RIL_YML1218 subpopulation, with the PVE ranging from 1.92% and 2.27%. The four QTLs detected in the RIL_Chang7-2 subpopulation distributed on chromosomes 1, 6, 8, and 9, with PVE ranging between 2.85% to 8.80%. Two QTLs located on chromosomes 8 and 10 were detected in the RIL_Q11 subpopulation, explaining 3.65% and 4.18% of the phenotypic variation. Finally, two QTLs detected on chromosomes 5 and 9 in the RIL_YML226 subpopulation with the QTL on chromosome 5 exhibiting the highest PVE (9.28%) among all the QTLs (Table 4).

**Table 3:** SNPs significantly associated with NLB resistance identified by GWAS in the MPP

List	Chr	SNP	REF	ALT	Effect	*P*(-log10)
1	1	1-43,673,103	C	T	− 0.56	13.69
2	1	1-119,902,290	C	T	− 0.45	8.97
3	1	1-254,624,609	A	G	− 0.42	9.01
4	2	2-186,686,058	T	A	− 0.59	12.48
5	2	2-220,965,609	T	G	− 0.26	8.93
6	3	3-5,145,248	T	G	0.38	10.87
7	3	3-28,397,163	T	C	0.87	10.39
8	3	3-116,657,860	C	A	0.62	10.68
9	3	3-140,159,846	A	G	− 0.41	10.93
10	4	4-228,167,118	A	G	− 0.30	9.01
11	5	5-49,193,921	C	A	0.63	8.50
12	5	5-203,356,810	C	A	0.34	8.77
13	6	6-105,594,656	AC	A	− 0.41	11.49
14	7	7-170,632,888	T	C	0.28	9.40
15	8	8-41,446,817	C	T	0.44	10.91
16	9	9-1,652,748	G	A	− 0.53	19.38
17	9	9-11,183,458	T	C	0.33	9.81
18	9	9-121,083,890	G	A	0.43	10.63

**Table 4:** List of QTLs linked to NLB resistance identified by QTL mapping of the four subpopulations

QTLs	Pop	Chr	LOD	Start marker	End marker	PVE	Add. effect
*qtl_YML1218_1*	YML1218 × Ye107	3	4.58	3-110,417,041	3-111,447,023	1.92	1.15
*qtl_YML1218_2*	YML1218 × Ye107	4	4.23	4-223,804,398	4-224,039,187	2.27	0.88
*qtl_Chang7-2_1*	Chang7-2 × Ye107	1	4.76	1-33,648,629	1-33,757,766	2.85	1.35
*qtl_Chang7-2_2*	Chang7-2 × Ye107	6	4.55	6-159,104,439	6-159,136,995	6.17	1.57
*qtl_Chang7-2_3*	Chang7-2 × Ye107	8	4.32	8-23,569,656	8-24,556,368	5.63	1.22
*qtl_Chang7-2_4*	Chang7-2 × Ye107	9	6.18	9-120,053,378	9-121,703,657	8.80	1.65
*qtl_Q11_1*	Q11 × Ye107	8	4.00	8-164,167,551	8-164,549,806	4.18	1.36
*qtl_Q11_2*	Q11 × Ye107	10	4.90	10-38,265,719	10-39,796,351	3.65	1.69
*qtl_YML226_1*	YML226 × Ye107	5	13.13	5-50,399,863	5-51,448,314	9.28	2.32
*qtl_YML226_2*	YML226 × Ye107	9	4.61	9-10,384,352	9-12,497,955	2.56	1.04

**Fig. 3:**
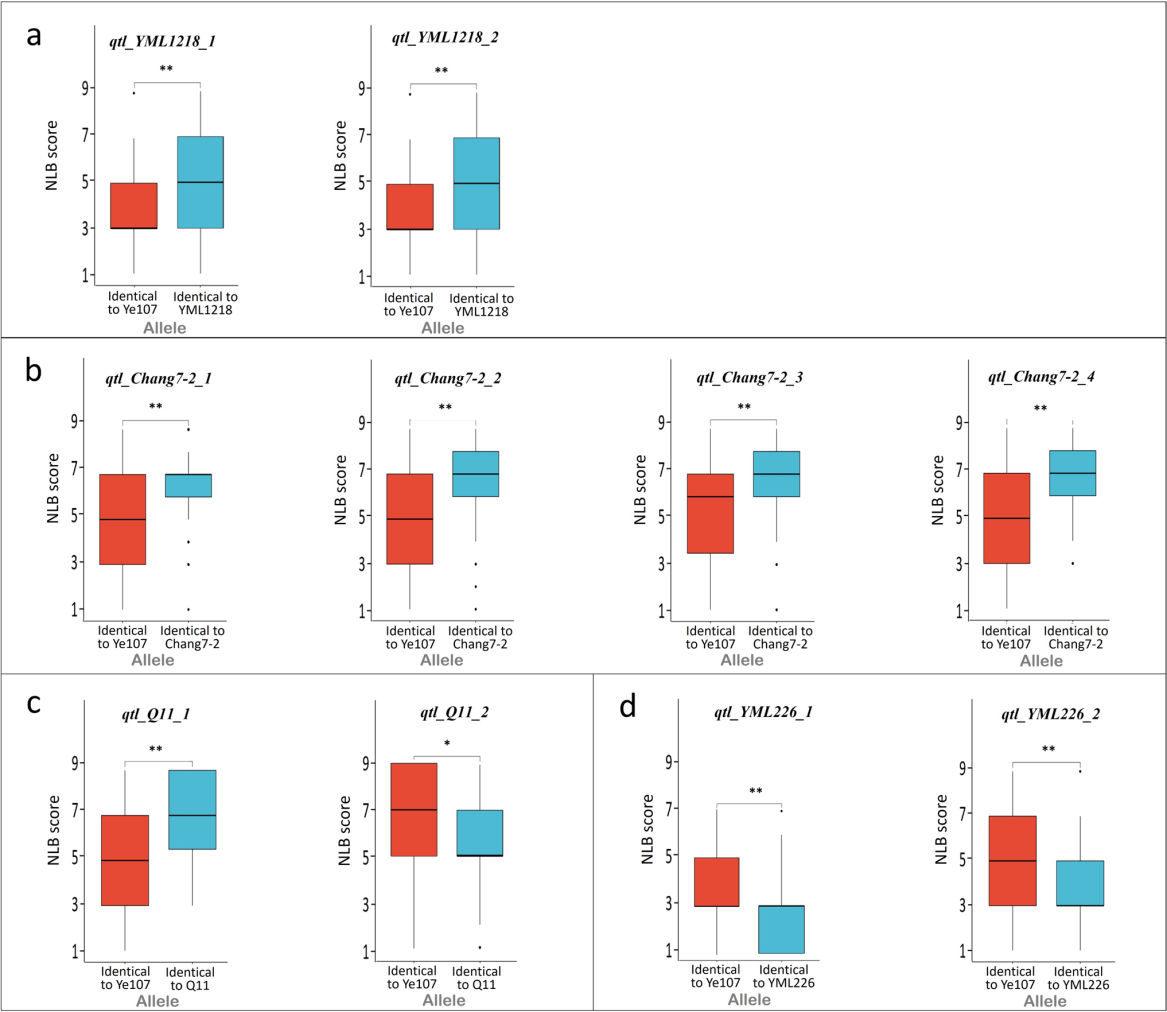
The NLB disease score for different alleles of each QTL. **a**, **b**, **c** and **d** exhibits QTLs detected in RIL_YML1218, RIL_Chang7-2, RIL_Q11 and RIL_YML226 subpopulations, respectively. * Indicates *P* < 0.005, and ** indicates *P* < 0.001

Analysis of the level of NLB resistance for families from each subpopulation revealed that, nine QTLs, except *qtl_Q11_2*, demostrated that families with the identical allele to the parental line with stronger NLB resistance exhibited significantly higher NLB resistance than those with the identical allele to the parental line with weaker NLB resistance (Fig. 3). These results indicate that, excluding *qtl_Q11_2*, the other nine QTLs had positive effects on NLB resistance, whereas *qtl_Q11_2* displayed a negative effect (Fig. 3c).

Upon comparing the results of QTL mapping for the four RIL subpopulations with the GWAS results of MPP, it was observed that SNP 9-11,183,458 and 9-121,083,890 overlapped with the intervel of *qtl_YML226_2* and *qtl_Chang7-2_4*, respectively. In addition, SNP 5-49,193,921 was found to be located at a physical distance of less than 1 Mb from *qtl_YML226 _*1. Therefore, it was speculated that *qtl_YML226 _*1 and SNP 5-49,193,921 were co-located (Fig. 2). Among the three co-located loci detected by GWAS and linkage analysis, SNP 5-49,193,921 was associated with the gene *Zm00001d014471*, which encodes a PPR superfamily protein. SNP 9-11,183,458 was associated with genes *Zm00001d045088*, *Zm00001d045013*, and *Zm00001d045014*, which encode pollen receptor-like kinase, pollen receptor-like kinase, and a PPR protein, respectively (Table 5). Notably, some of these proteins, such as LRR-RLK, are involved in plant disease resistance (Gomez and Boller, 2000; Wang et al. 2022). Therefore, the four genes identified in this study are likely to play a role in controlling NLB resistance in maize.

**Table 5:** Selected candidate genes responsible for NLB resistance identified through GWAS and overlapping the QTL regions

SNPs	*p* value (-log10)	Ref	Alt	Effect	Co-localized gene	Functional annotation
5-49,193,921	8.50	C	A	0.63	*Zm00001d014471*	Pentatricopeptide repeat (PPR) protein
9-11,183,458	9.81	T	C	0.33	*Zm00001d045088*	Leucine-rich repeat receptor-like kinase
					*Zm00001d045013*	Pollen receptor-like kinase
					*Zm00001d045014*	Pentatricopeptide repeat (PPR) superfamily protein

**Fig. 4:**
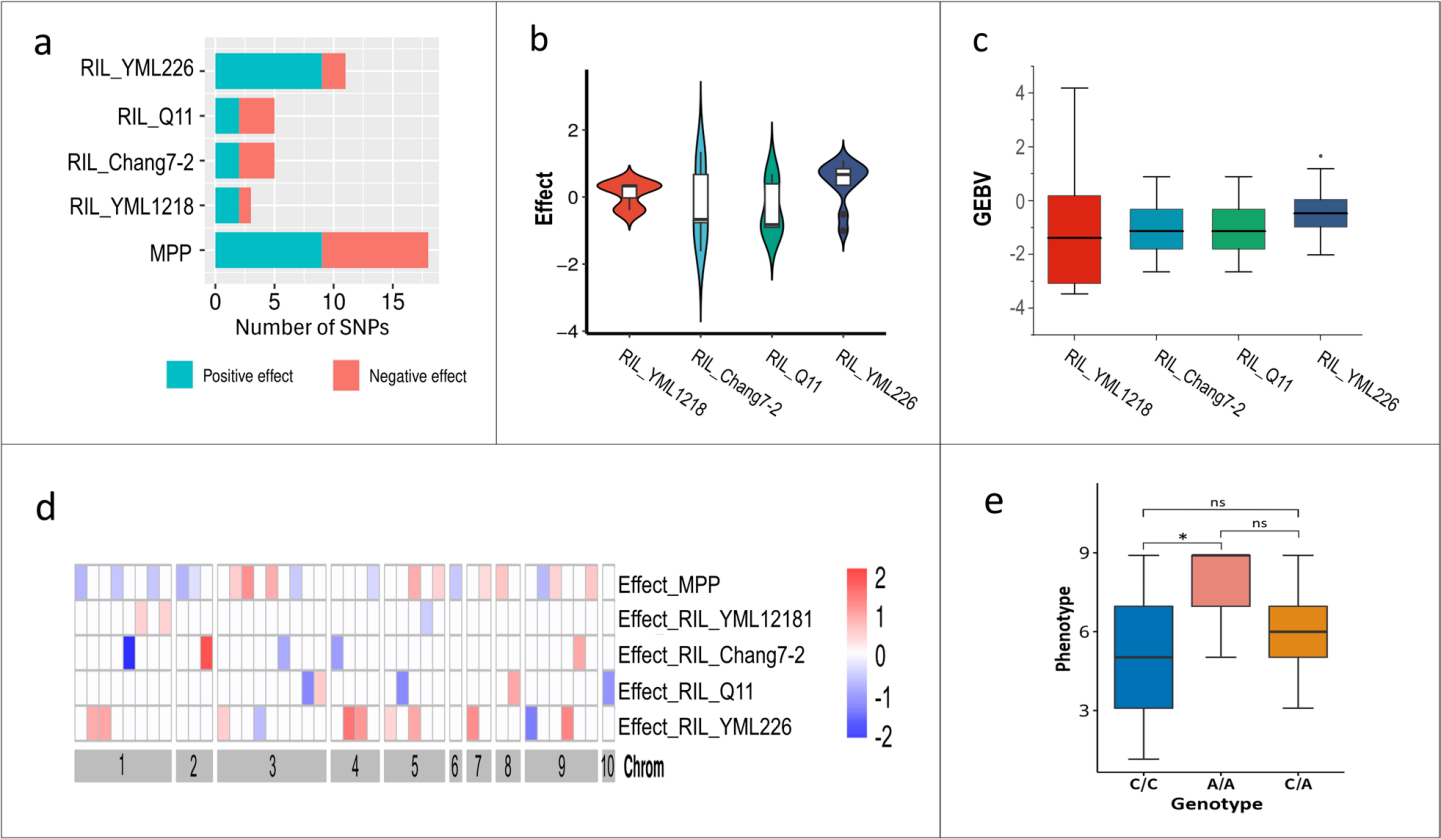
The variation, distribution and effects of SNPs in four RIL subpopulations. **a** Number of SNPs associated with NLB resistance in four subpopulations and MPP. **b** Distribution of SNP effect values for NLB resistance in four subpopulations. The white boxes in middle indicate the Q_25_ and Q_75_ quartile, while the black transverse lines within the white boxes indicate the median. The width of the violin plot indicates the density of SNPs. **c** Distribution of GEBV in the four subpopulations. The upper and lower sides of the boxes indicate the Q_25_ and Q_75_ quartile, while the black transverse lines within the boxes indicate the median. **d** Comparison of SNP distribution and effect values for NLB resistance between MPP and four RIL subpopulations. **e** The three haplotype of SNP 5-49,193,921 and their correlation with NLB resistance in the MPP. The y-axis represents the NLB disease score, while the x-axis represents the three haplotypes. Haplotype C/C significantly decrease the disease score, indicating higher NLB resistance

To determine the effects of the SNPs associated with NLB resistance in each RIL subpopulation, GWAS was performed separately for the four subpopulations. The results showed that a total of 24 SNPs significantly associated with NLB resistance were detected across the four subpopulations. Among these, eleven SNPs were detected in the RIL_YML226 subpopulation, accounting for 45.9% of all SNPs. In addition, five SNPs were detected in both the RIL_Chang7-2 and RIL_Q11 subpopulations, whereas only three SNPs were detected in the RIL_YML1218 subpopulation (Fig. 4a, Supplementary Table 3). It was observed that the NLB resistance level of the RILs was closely related to the number of SNPs with positive effects (Figs. 1c & 4b). By calculating the genomic estimated breeding value (GEBV), it was found that the RIL_YML226 subpopulation exhibited the highest GEBV among the four subpopulations (Fig. 4c), indicating that the SNPs associated with NLB resistance identified from the RIL_YML226 subpopulation not only had a high effect, but also had the strongest heritability.

Through the individual GWAS analysis of the four subpopulations, it was found that among the eleven SNPs significantly associated with NLB resistance in the RIL_YML226 subpopulation, nine had positive effects, accounting for 81.9% of the total SNPs (Fig. 4d). These positive SNPs led to the RIL_YML226 subpopulation exhibiting the highest NLB resistant level among all the subpopulations (Fig. 1c). Therefore, epistatic effects of the eleven SNPs associated with NLB resistance identified in the RIL_YML226 subpopulation were calculated. The results showed that there were eight pairs of SNPs with significant epistatic effects (*P* < 0.05) (Fig. 2c, Table 6, Supplementary Table 4). Notably, SNP 5-49,193,921 showed significant epistatic interactions with 1-101,177,576 and 3-3,709,493, contributing to 3.03% and 1.60% of the PVE, respectively. However, the PVE of the epistatic effect was relatively small compared to the QTL effect, which suggested that the epistatic effect had a smaller impact on NLB resistance.

**Table 6:** Significant epistatic interactions identified in the RIL_YML226 subpopulation

Chr1	SNP1	Chr2	SNP2	*P*.value(-log10)	PVE
1	1-71,337,126	3	3-86,873,356	3.41	6.95%
1	1-101,177,576	4	4-163,064,765	1.68	1.72%
1	1-101,177,576	5	5-49,193,921	3.02	3.03%
1	1-101,177,576	9	9-888,125	2.06	2.41%
3	3-3,709,493	5	5-49,193,921	1.44	1.60%
4	4-140,923,859	4	4-163,064,765	1.33	3.34%
4	4-140,923,859	9	9-888,125	1.32	1.90%
7	7-92,308,391	9	9-11,706,790	1.53	2.19%

Furthermore, the GWAS and QTL mapping results of the four RIL subpopulations were compared, revealing that in the RIL_Q11 and RIL_YML226 subpopulations, one and two SNPs, respectively, were detected through GWAS and overlapped within the QTL intervals (Supplementary Table 3). Among these, SNP 10-40,362,913 detected by GWAS in the RIL_Q11 subpopulation had a negative effect on NLB resistance, consistent with the performance of *qtl_Q11_2* in the subpopulation (Figs. 3c & 4d, Supplementary Table 3). In other words, families of the RIL_Q11 subpopulation carrying the identical allele to Q11 at this locus, were more resistant to NLB than families carrying the  allele identical to the moderately resistant parental line Ye107 (Figs. 3c & 4d). In addition, only SNP 5-49,193,921 identified in the RIL_YML226 subpopulation was consistent with the GWAS results conducted individually in the RIL subpopulations, MPP, and QTL mapping. This suggests that this locus may be a reliable candidate site for NLB resistance. To validate this,the haplotypes of the SNP 5–49,193,921 were analyzed. The results showed that this locus had three haplotypes, and compared with haplotypes A/A and C/A, haplotype C/C significantly enhanced NLB resistance in MPP (Fig. 4e). SNP 5-49,193,921 was associated with the gene *Zm00001d014471* (located at 49,402,864–49405873 bp on Chr5), which encodes a PPR protein related to post-transcriptional regulation of organelle genes (Barkan and Small 2014). Therefore, this SNP is considered an important molecular marker associated with NLB resistance, and its associated gene, *Zm00001d014471*, was identified as a key candidate gene for NLB resistance in this study.

### SNP variations in five parental lines

**Fig. 5:**
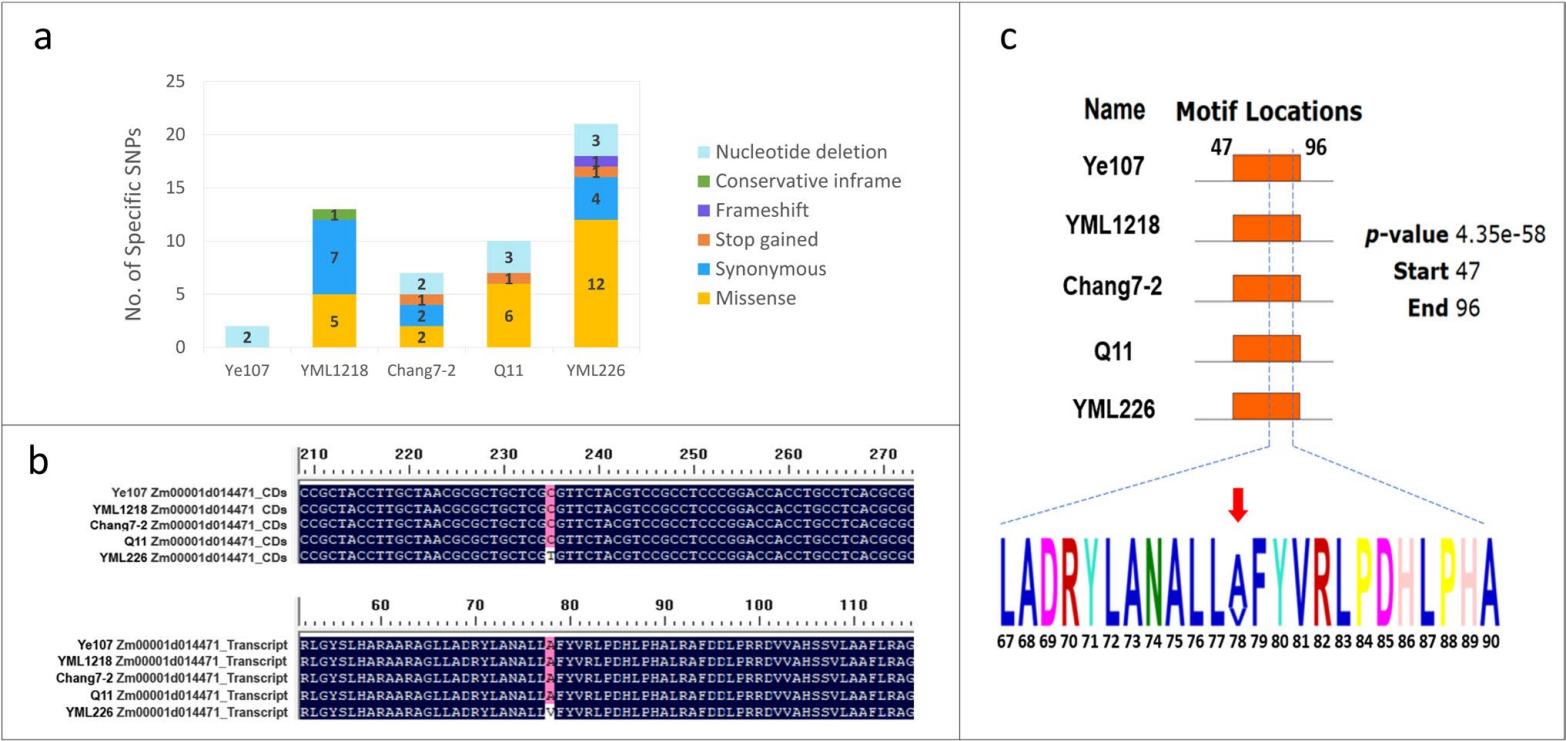
**a** Distribution of different types of specific SNPs in five parental lines. **b** SNP (upper) and amino acid (lower) variation in the parental line YML226. **c** Amino acid variation in the motif of PPR protein in five parental lines

The types and numbers of SNPs in the coding region of *Zm00001d014471* in the five parental lines were analyzed, and the specific SNP for each parental line were counted (Fig. 5a, Supplementary Table 5). The results indicated that the common parent Ye107 had only two nucleotide deletions and no specific non-synonymous or terminator SNPs in the coding region of the candidate gene *Zm00001d014471*. In contrast, YML226 exhibited the largest number of specific SNPs (21 SNPs), including 12 non-synonymous mutations (Fig. 5a, Supplementary Table 5). This suggested that the tropical line YML226 derived from the CATETO-related germplasm has a diverse genetic background compared to that of the temperate maize germplasm, which may explain the identification of 11 SNPs in the RIL_YML226 subpopulation through GWAS. Furthermore, by comparing the sequences of the five parental lines, a specific C/T mutation was found at the 235th position in YML226, resulting in a change in the 79th amino acid (from alanine to valine) (Fig. 5b). Analysis revealed that this amino acid mutation altered the protein motif (Fig. 5c). Consequently, we speculated that the nucleotide mutation at position 235 in the coding region of *Zm00001d014471* in YML226 changed the PPR protein structure, significantly enhancing NLB resistance in YML226.

### Relative expression of candidate gene *Zm00001d014471*

**Fig. 6:**
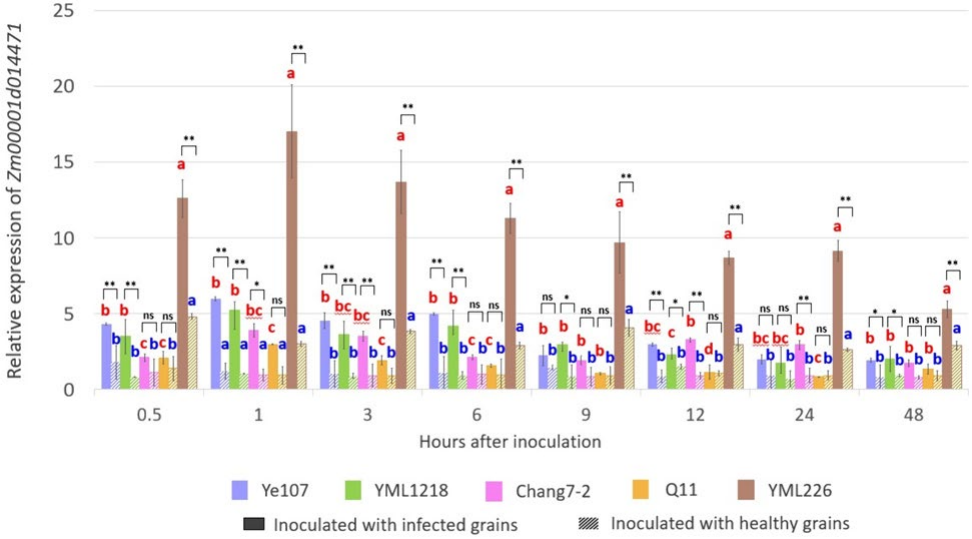
The relative expression level of *Zm00001d014471* in five parental lines at 0.5 to 48 h post inoculation. The red and blue lowercase letters above the corresponding columns represent the significance level at *P* < 0.05 for the inoculated and uninoculated samples at the sampling point. The symbols ns, *, and **indicate no significance, significance at *P* < 0.05, and significance at *P* < 0.01, respectively, between the inoculated and the uninoculated samples (color figure online)

To confirm the effect of the candidate gene *Zm00001d014471* on NLB resistance, the relative expression of *Zm00001d014471* in the five parental lines was measured through qRT-PCR. Leaf samples were collected from both inoculated and uninoculated plants at 0.5, 1, 3, 6, 9, 12, 24, and 48 h after artificial inoculation. The results showed that in the inoculated samples, the expression of *Zm00001d014471* in YML226 significantly increased, particularly within 24 h after inoculation (Fig. 6). Notably, even in uninoculated samples, YML226 displayed a significant difference in the expression of the *Zm00001d014471* gene compared to other parental lines, except at 1 h after inoculation*.* Of the inbred lines examined, YML226, which was selected from the CATETO germplasm,and contained the largest number of specific SNPs, exhibited the highest expression of the candidate gene *Zm00001d014471*. We hypothesized that the high expression of candidate gene *Zm00001d014471* in YML226 was caused by the mutation as above described, and led to the strongest resistance against NLB in the field (Figs. 5 & 6).

## Discussion

### Mapping of candidate gene *Zm00001d014471* for NLB resistance

In this study, the two commonly used methods for detecting functional loci that regulate quantitative traits: linkage mapping and association analysis, were used. In total, 10 QTLs and 18 SNPs were detected by linkage mapping and GWAS, respectively. Among these, SNP 5-203,356,810 was located within the QTL interval of previously identified QTL *qNCLB5.06* (Wang et al., 2018), while SNP 2-220,965,609 was in close proximity to gene Zm00001d007056 (located on 220,976,686-220,979,940 bp on chromosome 2 of the B73_V4 reference genome) identified by Thatcher et al. (2023). The exact or close proximity of the SNPs to previous studies indicated that validity of this study. However, both linkage mapping and association analysis have limitations in their application (Tanksley 1993; Campbell et al. 2005; Li et al. 2007), and combining the two methods may help to identify candidate functional loci, which may exploit the advantages of each method, thereby enhancing the reliability of mapping results. After combining the results of GWAS and linkage analysis, the loci *qtl_YML226_1* and SNP 5-49,193,921 were consistently identified in the YML226 subpopulation. Among all QTLs, *qtl_YML226_1* displayed the highest PVE (9.28%) while SNP 5-49,193,921 was identified at a *P*(-log10) of 8.5 through GWAS, indicating a high confidence in this new locus. The candidate gene *Zm00001d014471* associated with this locus may play a key role in NLB resistance in maize.

As a second-generation mapping population with abundant genetic variations and a clear genetic structure, MPP offers incomparable advantages over bi-parental or natural populations. Several multi-parent populations, such as nested association mapping (NAM), multi-parent advanced generation inter-cross (MAGIC), random-open-parent association mapping (ROAM), and complete-diallel design plus unbalanced breeding-like inter-cross (CUBIC) populations, have been utilized to analyze important traits in maize (Buckler et al. 2009; Kump et al. 2011; Pan et al. 2017; Liu et al. 2020). Dell’Acqua et al. (2015) observed that the number of rare alleles increased with the number of families in the mapping population, leading to a rapid decay of population LD, and significantly improving the efficiency of mapping target traits. However, our study revealed that the selection of parental lines significantly affected mapping efficiency. A GWAS of MPP and four RIL subpopulations indicated that while the number of SNPs significantly associated with NLB resistance identified in MPP was the highest, the number of SNPs with positive effects was the same as that detected in the RIL_YML226 subpopulation (Fig. 4a). This suggested that GWAS can identified candidate loci with positive effects when the mapping population contains fewer redundant lines. Notably, GWAS analysis of MPP identified a higher number of SNPs with negative effects, possibly due to the inclusion of RIL_Chang7-2 and RIL_Q11 subpopulations with weaker NLB resistance (Fig. 1c).

### NLB resistance is a quantitative trait controlled by multiple loci

Previous studies have identified several functional (candidate) genes or loci associated with NLB resistance, including *PH4GP-Ht1*, the receptor-like kinase gene *ZmWAKRLK1* (*Htn1*), the remorin gene *ZmREM6.3*,the caffeoyl-CoA 3-O-methyltransferase gene *ZmCCoAMT2*, *GRMZM2G042920*, *GRMZM2G041774*, *GRMZM2G056564*, and the *qNCLB7.02* (Hurni et al. 2015; Jamann et al. 2016; Yang et al. 2017; Wang et al., 2018; Rizzardi et al., 2022; Zhang et al. 2022; Thatcher et al., 2023). These studies showed that NLB resistance in maize is a complex quantitative trait that is controlled by multiple genes. In the present study, we identified the candidate functional gene *Zm00001d014471*, which encodes a PPR protein that was widely distributed in plants and regulate plant growth and development by participating in RNA processing (Barkan and Small 2014). Previous research in maize has shown that mutation in the PPR gene can lead to deficiencies in chloroplast ribosomes, causing the seedlings to turn pale green or white and ultimately leading to death (Schmitz-Linneweber et al 2006; Beick et al. 2008). Additionally, it was found that *MPPR6* promotes the maturation and translation initiation of rps3 transcripts, and its mutations can lead to delays in cell development (Manavski et al. 2012). Mutations in the maize *PPR2263* gene resulted in growth restriction, shorter leaves, and longer growth periods (Sosso et al. 2012). The authors also observed that homologous genes of *PPR2263* have similar functions in *Arabidopsis* (Sosso et al. 2012). Moreover, PPR proteins have been shown to be involved in stress resistance mechanisms. For instance, the expression of *Zm00001d014471* was increased significantly in maize leaves infected with *Cercospora Zeina* (Swart et al. 2017). Furthermore, the expression of this gene was significantly enhanced in maize leaves after inoculation with *Bacillus anthracis* (Hoopes et al. 2019). Therefore, we hypothesized that *Zm00001d014471* plays a crucial role in enhancing the resistance to various leaf diseases in maize.

In this study, a total of 10 QTLs related to NLB resistance were identified, however, no common QTL was observed among the four subpopulations. This could be due to the wide genetic variations present in the four different parental lines, resulting in distinct genetic effects of alleles among the four subpopulations.Nine of the ten QTLs exhibited positive effects on NLB resistance, with the exception of *qtl_Q11_2* in the RIL_Q11 subpopulation (Fig. 3). The presence of *qtl_Q11_2* revealed that alleles from the susceptible parental line may contribute to increased resistance. Xu et al. (2021) identified a specific fragment that was resistant to maize gray spot disease from the susceptible parent Q11. This demonstrates the complexity of the genetic mechanisms underlying the quantitative traits of plants. Additionally, among the nine SNPs with positive effects on NLB resistance identified in the subpopulations, none displayed a large individual effect (Table 3). This may be due to the fact that some QTLs in RIL subpopulations were homozygous after multi-generation recombination and linkage, which affected the epistatic effects of the loci, resulting in amplifying the phenotypic variation caused by the genetic effects. Therefore, the heritability of the QTL was increased even with relatively small PVE. In the RIL_YML226 subpopulation, the cumulative effects of these SNPs resulted in the highest NLB resistance among the four RIL subpopulations (Fig. 2c). These finding emphasize the significance of additive effects in improving NLB resistance in maize, suggesting that aggregating multiple NLB-resistant loci through marker-assisted breeding can effectively improve NLB resistance in maize.

### Trolical inbred line YML226 exhibited stronger resistance

Currently, most of the studies on NLB resistance in maize have used temperate germplasms, which generally exhibit narrow genetic variation and fewer favorable alleles than tropical and subtropical germplasms. However, most disease resistant genes exist in tropical inbred lines due to the temperature and humidity in tropical climates favor the occurrence and maintenance of disease resistant genes (Zhu et al., 2021). The inbred line YML226 was selected from the CATETO related germplasm originating from tropical regions in Brazil and Argentina (Fan et al. 2018). Hu et al. (2024) demonstrated that YML226 showed high resistance to maize gray spot disease. Subedi et al. (2015) conducted a survey of maize lines used in Nepal and found that maize derived from the CATETO germplasm imported from Yunnan Province, China showed high leaf spot resistance and yield. Our study revealed that YML226 harbored the largest number of genetic variants (Fig. 5a), and the RIL_YML226 subpopulation contained the highest number of positive effect SNPs among all RIL subpopulations (Fig. 5a). Therefore, we speculate that the abundance of genetic variants in YML226 significantly contributes to NLB resistance. Moreover, these loci not only displayed large effect values but also high GEBV, suggesting that YML226, is an important resource for enhancing NLB resistance in temperate maize germplasms. Additionally, the reference genome used in this study was the temperate maize B73, which is widely used in maize research. As the genetic backgrounds of temperate and tropical maize are distinct, using B73 as the reference genome for identifying NLB-resistant genes in the tropical maize line YML226 may limit the identification of NLB-resistant genes. Hence, enhancing the genetic diversity of temperate maize germplasms and exploring NLB-resistant genes in tropical maize germplasms, such as YML226, remains crucial.

Our analysis of SNPs in the coding region of the *Zm00001d014471* gene across the five parental lines revealed that YML226 possesses the largest number of variants compared to the other parental lines. This abundance of genetic variants may lead to functional alterations in *Zm00001d014471* in YML226, thereby improving its resistance to NLB.

## Conclusion

In this study, *qtl_YML226_1* and SNP 5-49,193,921 were identified in an introgressed line, YML226, developed from temperate and tropical maize. The QTL explained 9.28% of the PVE, whereas the *P*(-log10) value of the SNP was 8.5, as identified through GWAS. Therefore, this candidate functional locus was considered to have a major effect on NLB resistance. The relative expression of *Zm00001d014471*in maize leaves, was positively correlated with NLB resistance. Therefore, *Zm00001d014471* was considered as a candidate functional gene with a positive effect on NLB resistance. Furthermore, we observed that YML226, derived from the CATETO germplasm, exhibited the highest number of genetic variants in the coding region of the candidate gene compared to other temperate maize lines. Among these variants, the mutation at position 235 caused amino acid changes, potentially contributing to enhanced resistance of YML226. This study has identified NLB resistance candidate loci that can be used in the molecular breeding for NLB resistance in maize, and can assist in the selection of resources resistant to NLB.

### Supplementary Information

Below is the link to the electronic supplementary material.

Supplementary file1 (XLSX 31 KB)

## Data Availability

The raw sequencing data have been deposited into the National Center for Biotechnology Information under accession number PRJNA967807.
